# Delving into tRNA-derived small RNAs in multiple myeloma: elevated 3′U-tRF^SerTGA^ leads to poor disease prognosis

**DOI:** 10.1038/s41416-026-03447-5

**Published:** 2026-05-04

**Authors:** Konstantinos Soureas, Panagiotis Malandrakis, Maria-Alexandra Papadimitriou, Ioannis Ntanasis-Stathopoulos, Katerina-Marina Pilala, Christine-Ivy Liacos, Maria Gavriatopoulou, Efstathios Kastritis, Meletios-Athanasios Dimopoulos, Andreas Scorilas, Evangelos Terpos, Margaritis Avgeris

**Affiliations:** 1 Department of Biochemistry and Molecular Biology, Faculty of Biology, National and Kapodistrian University of Athens, Athens, Greece; 2 Laboratory of Clinical Biochemistry—Molecular Diagnostics, Second Department of Pediatrics, School of Medicine, National and Kapodistrian University of Athens, “P. & A. Kyriakou” Children’s Hospital, Athens, Greece; 3 Department of Clinical Therapeutics, School of Medicine, National and Kapodistrian University of Athens, Alexandra General Hospital, Athens, Greece

**Keywords:** Myeloma, Tumour biomarkers, Prognostic markers, Small RNAs

## Abstract

**Background:**

Multiple myeloma (MM) is an incurable malignancy, marked by treatment resistance and frequent relapses, posing ongoing challenges to patients’ long-term management. Herein, we have examined tRNA-derived small RNA fragments (3′U-tRFs), generated from precursor tRNAs, to identify MM-related 3′U-tRFs in ameliorating MM precision prognostics.

**Methods:**

3′U-tRF profiles were generated by small RNA-seq data. Target prediction and gene ontology analysis were assessed by tRFtarget and DAVID databases, respectively. CD138 + 3′U-tRF^SerTGA^ levels were quantified in our MM screening cohort (*n* = 136 patients) by RT-qPCR. Kaplan–Meier and Cox proportional regression analyses were performed, using disease progression and patients’ mortality as clinical endpoints. Internal validation was conducted by bootstrap Cox regression while clinical benefit on patients’ prognosis was assessed by decision curve analysis (DCA).

**Results:**

Small RNA-seq data analysis highlighted the significantly increased 3′U-tRF^SerTGA^ levels in MM cell lines compared to normal cells (FC: 14.03). Our screening cohort confirmed the significantly higher risk for short-term progression and worse survival of the patients presenting elevated 3′U-tRF^SerTGA^. 3′U-tRF^SerTGA^-fitted multivariate models demonstrated superior risk-stratification of the patients for treatment response and prognosis.

**Conclusions:**

Our study indicate the elevated 3′U-tRF^SerTGA^ as a strong independent predictor of poor first-line chemotherapy outcomes and MM progression, providing refined stratification of patient risk.

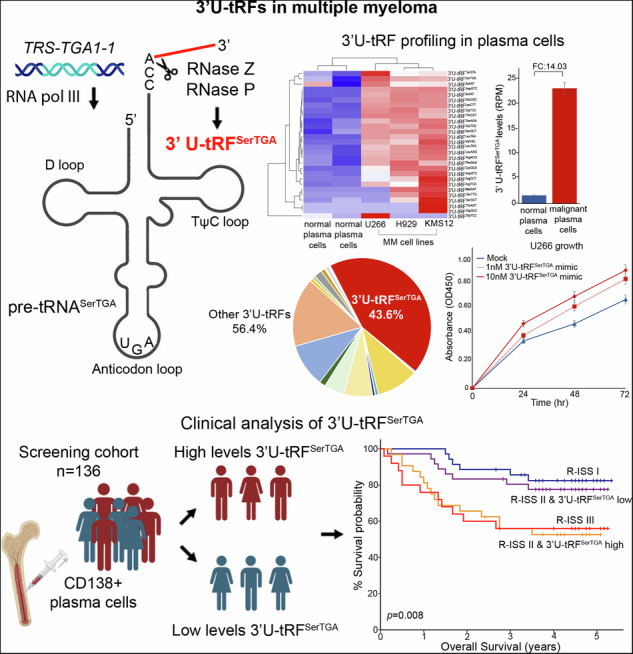

## Background

Multiple myeloma (MM) is the second most common haematological cancer following non-Hodgkin’s lymphoma and marked by the uncontrolled growth of plasma cells [1, 2]. MM typically originates from benign precursor states, specifically monoclonal gammopathy of undetermined significance (MGUS) and smoldering multiple myeloma (sMM), which may ultimately progress to symptomatic MM [2, 3]. From the clinical perspective, MM leads to bone marrow failure, bone lesions and hypercalcemia, remaining essentially incurable [2]. Currently, the clinically established stratification system is the Revised International Staging System (R-ISS), which stratifies patients based on the serum β2 microglobulin (B2M), serum lactate dehydrogenase (LDH) levels and cytogenetic abnormalities, improving treatment decisions [4, 5]. Although considerable efforts have been made towards the efficient prognosis and therapy of MM, patients eventually develop treatment resistance and relapse [6–9]. Hence, the improvement of patients’ stratification and ability to provide more personalised prognosis/therapy remain formidable challenges.

**Fig. 1:**
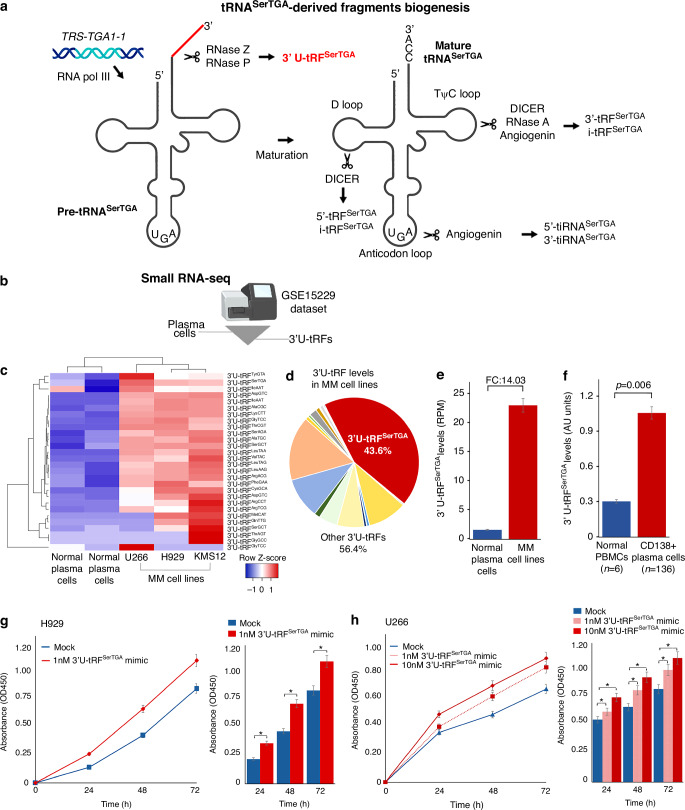
In silico analysis of 3′U-tRF^SerTGA^. **a** Graphical representation of tRFs biogenesis based on tRNA^SerTGA^. **b** Analysis of GSE15229 small RNA-seq dataset for 3′U-tRFs profiling in plasma cells. **c** Heatmap of the 3′U-tRFs levels in normal plasma cells and MM cell lines. **d** 3′U-tRF levels in MM cell lines. **e** 3′U-tRF^SerTGA^ levels in MM cell lines compared to normal plasma cells. **f** 3′U-tRF^SerTGA^ levels in CD138+ plasma cells (*n* = 136) compared to PBMCs from healthy donors (*n* = 6), Growth rate of 3′U-tRF^SerTGA^ transfected H929 (**g**) and U266 (**h**) cells.

Non-coding RNAs (ncRNAs) represent a swiftly growing class of regulatory RNAs, recognised as potent regulators of gene expression at the post-transcriptional and epigenetic levels [10–12]. In this context, tRNA-derived fragments (tRFs), also referred as tRNA-derived small RNAs (tsRNAs), are a recently identified class of small ncRNAs, typically 14–50 nucleotides in length, generated from both precursor and mature nuclear and mitochondrial tRNAs (Fig. 1a) [13–15]. tRFs from mature tRNAs are divided into five subtypes based on the cleavage site; 5′-tRFs, 3′-tRFs, 5′-tiRNAs, 3′-tiRNAs and internal tRFs (i-tRFs) [13–15]. The 5′-tRFs and 3′-tRFs are being formed by Dicer-dependent cleavage at the D-loop, and by Dicer- or angiogenin-dependent cleavage at the TψC-loop, yielding fragments from the 5′- or 3′-ends of mature tRNAs, respectively, while tRNA halves (5′-tiRNAs and 3′-tiRNAs) are stress-responsive fragments produced through angiogenin-mediated cleavage in the anticodon loop of mature tRNAs [16, 17]. Furthermore, i-tRFs arise through Dicer cleavage within the internal regions of mature tRNAs [13, 14]. Interestingly, 3′U-tRFs are generated within the nucleus following 3′-tail cleavage of pre-tRNAs from RNase Z, representing unique sequences that end with a stretch of 3 uracils (U) [14, 18, 19]. While tRFs initially considered as byproducts of tRNA metabolism, recent studies have highlighted their role in the regulation of gene expression and cellular homoeostasis, impacting in essential cellular processes, including proliferation, migration and apoptosis as well as their deregulated levels in numerous human pathologies, and most importantly cancer [14, 20].

Intriguingly, 3′U-tRFs are the least analysed family members and recent studies have highlighted their deregulated levels in multiple malignancies; however, their impact in MM realm is still missing. The aim of the present study was to investigate for the first time the involvement of 3′U-tRFs in MM and decipher their clinical value as novel clinical tools for patients’ prognosis and treatment decisions. Herein, analysing small RNA-seq data we have identified 3′U-tRF^SerTGA^, derived from pre-tRNA^SerTGA^, as the most abundant and highly upregulated 3′U-tRF in malignant compared to normal plasma cells, while 3′U-tRF^SerTGA^ significantly promoted growth rates of transfected MM cells. Moreover, the clinical evaluation of 3′U-tRF^SerTGA^ in our MM screening cohort (*n* = 136) highlighted the significantly worse prognosis and chemotherapy outcome of the MM patients with elevated 3′U-tRF^SerTGA^ levels in CD138+ plasma cells, independently of their clinicopathological data. Ultimately, 3′U-tRF^SerTGA^-fitted models leaded to improved risk-stratification and superior positive prediction of patient’s short-term progression and poor treatment response compared to clinically established disease markers, underlining 3′U-tRF^SerTGA^ impact in tailored MM management.

## Methods

### Screening cohort

The screening cohort of the study included 136 newly diagnosed treatment-naive MM patients, identified according to the International Myeloma Working Group (IMWG) criteria. Bone marrow (BM) aspirates were collected at diagnosis in the Department of Clinical Therapeutics, ‘Alexandra’ Hospital, Athens, Greece. At the initial assessment, patients underwent a baseline evaluation through blood, biochemical, and imaging tests. Cytogenetic abnormalities were detected using conventional cytogenetic techniques and interphase fluorescence in situ hybridisation (FISH) on BM aspirates from trephine biopsies, while bone disease was evaluated through whole-body low dose computed tomography (WBLDCT).

Of the 136 initially enroled patients, 101 (74.3%) received Bortezomib-Lenalidomide-Dexamethasone, while 21 (15.4%) and 11 (8.1%) were treated with Bortezomib-based and Lenalidomide-Dexamethasone, respectively. Based on R-ISS staging, 25.7%, 50.0% and 18.3% of patients were classified as R-ISS I, II and III, respectively, while bone disease was observed in 68.4% of patients at diagnosis. Treatment responses were monitored monthly following IMWG criteria using blood and urine tests. The study adhered to the ethical guidelines of the 1975 Declaration of Helsinki, as amended in 2008, and was approved by the Ethics Committee of ‘Alexandra’ Hospital. Informed consent was obtained from all participants prior to sampling. Patients’ clinicopathological features are provided in Table 1.

**Table 1:** Clinicopathological features of the screening cohort

Variable	No. of patients (*n* = 136)
**R-ISS stage**	
R-ISS I	**35** (25.7%)
R-ISS II	**68** (50.0%)
R-ISS III	**25** (18.3%)
Missing data	8
**R2-ISS stage**	
R2-ISS I (low-risk)	**22** (16.2%)
R2-ISS II (low/intermediate-risk)	**33** (24.3%)
R2-ISS III (intermediate/high-risk)	**60** (44.1%)
R2-ISS IV (high-risk)	**13** (9.6%)
Missing data	8
**ISS Stage**	
ISS I	**42** (30.9%)
ISS II	**39** (28.7%)
ISS III	**53** (39.0%)
Missing data	2
**Prior sMM/MGUS**	
Yes	**20** (14.7%)
No	**115** (84.6%)
Missing data	1
**Gender**	
Male	**75** (55.1%)
Female	**61** (44.9%)
**Therapy**	
Bortezomib-Lenalidomide-Dexamethasone	**101** (74.3%)
Bortezomib-based treatment	**21** (15.4%)
Lenalidomide-Dexamethasone	**11** (8.1%)
Other	1 (0.7%)
Not complete treatment	1 (0.7%)
**Bone disease**	
Yes	**93** (68.4%)
No	**35** (25.7%)
Missing data	8
**HDM/ASCT**	
Yes	**41** (30.1%)
No	**95** (69.9%)
**B2M**	
<5.5 mg/l	**83** (61.0%)
>5.5 mg/l	**52** (38.2%)
Missing data	1
**LDH**	
≤220 U/l	**105** (77.2%)
>220 U/l	**31** (22.8%)
**Marrow plasma cells**	
<60%	**60** (44.1%)
≥60%	**76** (55.9%)
**Response to first**^**-**^**line therapy**	
sCR, CR, VGPR	**92** (67.6%)
PR, SD, PD	**43** (31.6%)
Missing data	1
**Disease monitoring**	
Follow-up patients	**136**
Relapse	**54** (39.7%)
Death/Alive	**44** (32.4%)/**92** (67.6%)
Progression/Progression-free survival	**76** (55.9%)/**60** (44.1%)

Bold text is used to denote category headers and primary data subgroups.

*HDM/ASCT* high dose melphalan therapy with autologous stem cell transplantation, *sCR* stringent complete response, *CR* complete response, *VGPR* very good partial response, *PR* partial response, *SD* stable disease, *PD* progressed disease.

### Small RNA-seq in silico analysis and Gene Ontology (GO) analysis

Small RNA-seq normalised data of GSE15229 GEO dataset were retrieved through tRFdb database (https://app05.bioinformatics.virginia.edu/index.php) [21, 22]. To predict 3′U-tRF^SerTGA^ targets, RNAhybrid and IntaRNA tools were used, through the tRFtarget 2.0 database (http://trftarget.net/) [23]. Thereafter, specific inclusion criteria, includingbinding regions at 3′-UTR, free energy ≤−16 Kcal and maximum complementary length >8 were applied in order to minimise prediction error rates. The target gene set was functionally annotated through the Database for Annotation, Visualisation and Integrated Discovery (DAVID; https://david.ncifcrf.gov/tools.jsp) for Gene Ontology (GO) enrichment analysis of biological processes (BPs), cellular components (CCs) and molecular functions (MFs) [24].

### Cell culture

Human myeloma cell lines U266, NCI-H929, RPMI-8226 and the immortalised healthy B lymphoblastoid cell line RPMI-1788 were used for 3′U-tRF^SerTGA^ levels quantification, while U266 and NCI-H929 were used for in vitro functional studies. All cell lines were cultured in RPMI-1640 medium (Biosera, France) supplemented with 10% FBS (PAN-Biotech GmbH, Germany), 2 mM L-glutamine (Biosera) and 1% penicillin/streptomycin solution (Biosera). Cell lines were maintained *per* manufacturer instructions at 37 °C in humid atmosphere of 5% CO_2_.

### Transfection

For 3′U-tRF^SerTGA^ transfection, Lipofectamine RNAiMAX reagent (Invitrogen, Carlsbad, CA, USA) was used according to manufacturer’s instructions. Briefly, 60.000 cells were seeded in 24-well plates and cultured without antibiotics addition. The following day, cells were transfected with 1 and 10 nM 3′U-tRF^SerTGA^ mimic (5′–CGGAAGCGGGUGCUCUUAUUU–3′) in triplicates. Mock cells (w/o mimic) were used as controls. 48 h later, cells were harvested, total RNA was extracted and 3′U-tRF^SerTGA^ levels were measured by RT-qPCR for transfection efficiency.

### Cell viability assay

Cell Counting Kit 8 (WST-8/CCK8) assay was used to test the cell viability and growth of the cells. Cells were harvested 16 h post-transfection and 10^4^ of U266 and NCI-H929 cells were seeded into 96-well plates in complete growth medium. Plating was performed in triplicate with one plate for each time point. At timepoints 0 h, 24 h, 48 h and 72 h, 10 μl of CCK8 solution (Abcam, Cambridge, UK) was added and incubated at 37 °C for 2 h, following gently shaking. Absorbance was read at 450 nm on a HumaReader HS microplate reader (HUMAN, Wiesbaden, Germany).

### Apoptosis and necrosis assay

RealTime-Glo Annexin V Apoptosis and Necrosis Assay (Promega, Madison, USA) was used to examine the apoptotic and/or necrotic impact of 3′U-tRF^SerTGA^ mimic via the measurement of annexin V binding and a pro-fluorescent DNA dye, respectively. Briefly, 10^4^ of U266 and NCI-H929 cells were seeded into 96-well white plates, and the assay was performed *per* manufacturer instructions. At timepoints 0 h, 24 h, 48 h and 72 h, luminescence (apoptosis) and fluorescence (necrosis) measurements were snapped, using Spark Multimode Microplate Reader (Tecan Life Sciences, Zurich, Switzerland).

### CD138+ plasma cells isolation and RNA extraction

BM aspirates were collected in EDTA tubes, and mononuclear cells were separated via Ficoll-Paque density gradient centrifugation. CD138+ plasma cells enrichment was achieved using magnetic cell sorting (MACS) with immunomagnetic microbeads conjugated with anti-CD138 monoclonal antibodies (Miltenyi-Biotec GmbH, Bergisch Gladbach, Germany). Additionally, peripheral blood mononuclear cells (PBMCs) from healthy donors (*n* = 6) were isolated, immediately after the sample collection, by Ficoll-Paque density gradient centrifugation and washed twice with phosphate-buffered saline (PBS) containing no Ca/Mg.

Extraction of total RNA was followed using TRI-Reagent (Molecular Research Center, Cincinnati, OH, USA), according to manufacturer’s instructions. Total RNA was dissolved in RNA Storage Solution (Ambion, Austin, TX, USA), and its concentration was measured with Qubit 2.0 Fluorometer (Invitrogen).

### RNA 3′-end polyadenylation and first-strand cDNA synthesis

200 ng of total RNA was polyadenylated at the 3′-end by 1 U of *E. coli* poly(A) polymerase (New England Biolabs Inc., Ipswich, MA, USA) and 800 μM ATP in a 10 μL reaction at 37 °C for 60 min. Enzyme inactivation was carried out at 65 °C for 10 min. Subsequently, polyadenylated RNA served as template for first-strand cDNA synthesis in a 20 μL reaction, using 200 U M-MLV Reverse Transcriptase (Invitrogen), 40 U RNaseOUT Recombinant Ribonuclease Inhibitor (Invitrogen), 10 mM dNTP Mix and 250 nM oligo-dT adaptor (5′-GCGAGCACAGAATTAATACGACTCACTATAGGTTTTTTTTTTTTVN-3′, where V represents G, A, C and N represents G, A, T, C). Reverse transcription took place at 37 °C for 60 min followed by enzyme inactivation at 70 °C for 15 min.

### Quantitative real-time PCR (qPCR)

Quantitative real-time PCR (qPCR) assays, using SYBR Green I dye, were developed and conducted to quantify 3′U-tRF^SerTGA^ levels. Specific primers were designed for the small nucleolar RNA C/D box 48 (SNORD48; also known as RNU48; 5′-TGATGATGACCCCAGGTAACTCT-3′) and 3′U-tRF^SerTGA^ (5′-CGGAAGCGGGTGCTCTTA-3′) according to published RNA sequences (NCBI RefSeq: NR_002745.1 for SNORD48 and RNAcentral ID: URS0000092302_9606). The specific forward primers and a universal reverse primer (5′-GCGAGCACAGAATTAATACGAC-3′), complementary to the above-mentioned oligo-dT adaptor, were used for the amplification of a 64 bp and 105 bp amplicons for 3′U-tRF^SerTGA^ and RNU48, respectively. The qPCR reactions were performed in 7500 Real-Time PCR System (Applied Biosystems, Carlsbad, CA, USA), consisted of Kapa SYBR Fast Universal 2X qPCR Master Mix (Kapa Biosystems, Inc., Woburn, MA, USA), 200 nM of each primer, and 1 ng of cDNA template in 10 μL total volume. The cycling protocol included polymerase activation at 95 °C for 3 min, followed by 40 cycles of 95 °C for 15 s for denaturation and 60 °C for 1 min for primer annealing and extension. To ensure reproducibility, all reactions were conducted in duplicate. Melting curve analysis and agarose gel electrophoresis were performed following amplification to evaluate products’ specificity. Quantification of 3′U-tRF^SerTGA^ levels was conducted by the 2^−ΔΔCT^ method, using RNU48 as the reference control for normalisation purposes.

### Statistical analysis

Statistical analyses were performed using IBM SPSS Statistics version 20 (IBM Corp., Armonk, NY, USA). Student’s *t* test was applied in CCK8 data to evaluate the differences between the tested groups. The X-tile algorithm was used to select the optimal cut-off values of 3′U-tRF^SerTGA^ levels. Survival analysis was conducted by Kaplan–Meier curves and Cox proportional regression analysis, using disease progression (relapse and/or death; whichever came first) and patients’ mortality as clinical end-point events for progression-free (PFS) and overall survival (OS) respectively. Bootstrap analysis was performed for internal validation of Cox regression models. Ultimately, the clinical benefit of 3′U-tRF^SerTGA^ in relation to patient outcomes were assessed using decision curve analysis (DCA) as described by Vickers et al., via STATA version 13 (StataCorp LLC, College Station, TX, USA) [25].

## Results

### In silico analysis of 3′U-tRFs in plasma cells

The analysis of small RNA-seq data (GSE15229) highlighted 3′U-tRF^SerTGA^ as the most abundant 3′U-tRF (43.6%; Fig. 1c) and significantly overexpressed (Fold Change; FC = 14.03; Fig. 1d, e) in MM (cell lines: U266; GSM497063, H929; GSM497076, KMS12; GSM497067) compared to normal plasma cells (primary human plasma cells; GSM380325, GSM380329). Notably, 3′U-tRF^SerTGA^ displayed higher relative abundance in three MM cell lines (H929, U266 and RPMI-8226) than in a non-malignant RPMI-1788 (Supplementary Fig. 1), while 3′U-tRF^SerTGA^ levels were significantly higher in MM CD138+ plasma cells compared to healthy donors PBMCs (Fig. 1f).

To gain insight into the functional role of 3′U-tRF^SerTGA^, we performed target-prediction and GO enrichment analysis. RNAhybrid and IntaRNA target prediction tools were used to identify potential targets of 3′U-tRF^SerTGA^. At first, 5000 overlapping target-genes were retrieved from the two prediction tools. By applying specific incusion criteria to minimise prediction error rates, 884 genes were identified for further analysis as possible targets of 3′U-tRF^SerTGA^. GO analysis of target genes was performed with DAVID database, retaining 24 BPs, 8 CCs and 5 MFs terms with enrichment score >1.3, *p* < 0.05 and gene count >20 (Supplementary Fig. 2A, B). including localisation, regulation of signalling and cell communication. Ultimately, our in silico analysis, prompt us to further investigate the clinical value of 3′U-tRF^SerTGA^ in our MM screening cohort.

### 3′U-tRF^SerTGA^ enhances cell growth of myeloma cells

To strengthen our in-silico findings and to validate 3′U-tRF^SerTGA^ impact on myeloma cell growth, H929 and U266 cells were transfected with 1 nM and 10 nM 3′U-tRF^SerTGA^ mimic. Mock cells (w/o mimic) were used as controls. Exogenous 3′U-tRF^SerTGA^ resulted in significantly increased growth rate of both H929 and U266 cells, following 24 h, 48 h and 72 h of transfection (Fig. 1g, h). Strikingly, in U266 cells a dose-dependent manner was also observed. Regarding the apoptosis rates, a moderate reduction was observed in 3′U-tRF^SerTGA^ transfected cells; although not statistically significant.

### Elevated 3′U-tRF^SerTGA^ levels in CD138+ plasma cells are associated with patients’ short-term progression and poor survival

Initially, analysis of 3′U-tRF^SerTGA^ levels in CD138+ plasma cells of our screening cohort (*n* = 136) across genomically defined MM subgroups, pointed out its correlation with 17p13 deletion (*p* < 0.001; Supplementary Fig. 3A) and t(11;14) translocation (*p* = 0.002; Supplementary Fig. 3D). Notably, other cytogenetic abnormalities, including 13q deletion, 1q21 gain/amp and t(4;14) translocation presented non-significant results (Supplementary Fig. 3).

**Fig. 2:**
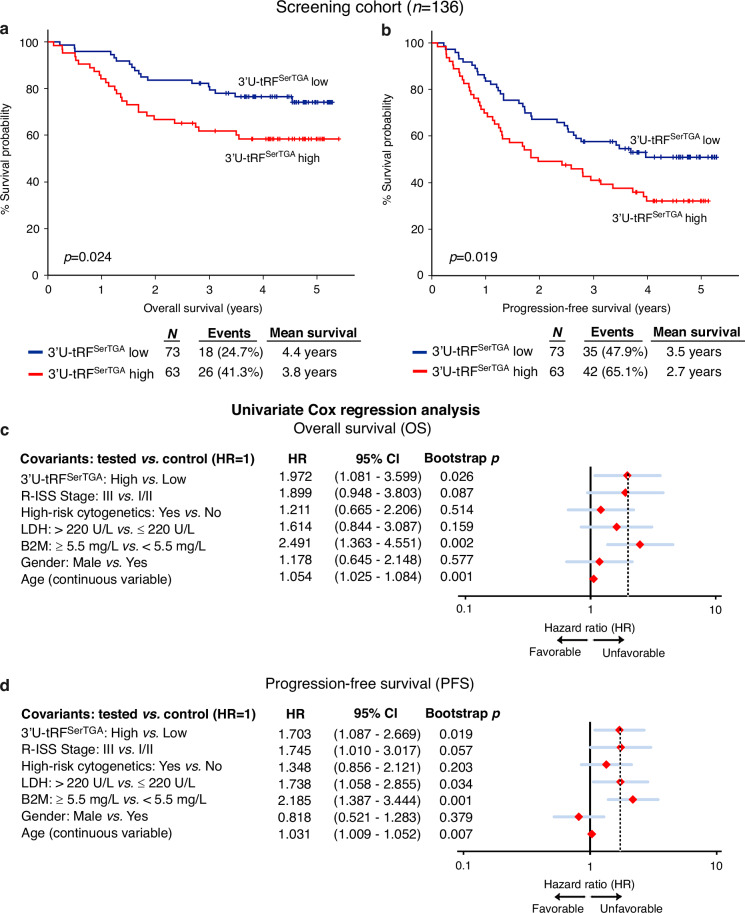
Increased 3′U-tRF^SerTGA^ levels in CD138 +  plasma cells at diagnosis are associated with poor overall and progression-free survival. Kaplan–Meier survival curves for OS (**a**) and PFS (**b**) of the screening cohort. *p*-values calculated by log-rank test. Forest plots of the univariate Cox proportional regression analysis for OS (**c**) and PFS (**d**). Internal validation was performed by bootstrap analysis based on 1000 bootstrap samples. HR Hazard Ratio, 95% CI 95% confidence interval of the estimated HR.

**Fig. 3:**
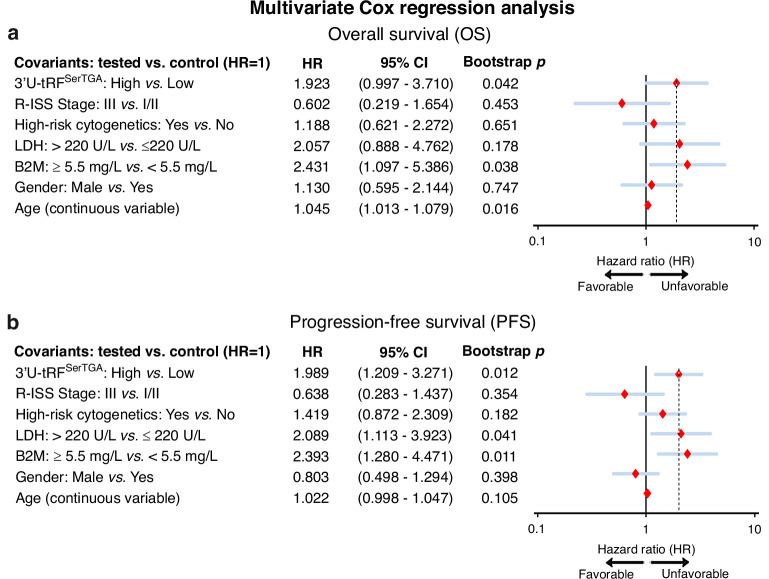
3′U-tRF^SerTGA^ levels in CD138+ plasma cells could independently predict patients’ post-treatment outcome. Forest plots of the multivariate Cox proportional regression analyses for OS (**a**) and PFS (**b**) of the patients. Multivariate analysis adjusted for 3′U-tRF^SerTGA^ levels in CD138+ plasma cells, R-ISS stage, high-risk cytogenetics, B2M, LDH, gender and age. Internal validation was performed by bootstrap analysis based on 1000 bootstrap samples. HR Hazard Ratio, 95% CI 95% confidence interval of the estimated HR.

Subsequently, regarding survival analysis, the 53rd percentile of 3′U-tRF^SerTGA^ levels was adopted as the optimal cut-off value (X-tile algorithm) to classify patients to ‘3′U-tRF^SerTGA^-high’ and ‘3′U-tRF^SerTGA^-low’ groups. In this regrad, Kaplan–Meier curves depicted the significantly shorter OS (*p* = 0.024, Fig. 2a) and PFS (*p* = 0.019; Fig. 2b) of the patients with increased 3′U-tRF^SerTGA^ levels in CD138+ plasma cells at diagnosis compared to ‘3′U-tRF^SerTGA^-low’ group. Moreover, univariate Cox regression analysis (Fig. 2c, d; Supplementary Table 1) confirmed the worse survival (HR = 1.972, 95% CI: 1.081–3.599, *p* = 0.026; Fig. 2c) and the higher risk for post-treatment progression (HR = 1.703, 95%CI: 1.087–2.669, *p* = 0.019; Fig. 2d) of the ‘3′U-tRF^SerTGA^-high’ patients. Finally, multivariate Cox regression models adjusted for R-ISS stage, high-risk cytogenetics [t(4;14), t(14;16), del(17p13), gain/amp(1q21)], LDH, B2M, gender and age (Fig. 3; Supplementary Table 1), clearly documented the independent prognostic value of the elevated 3′U-tRF^SerTGA^ levels in CD138+ plasma cells for the poor OS (HR = 1.923, 95%CI: 0.997–3.710, *p* = 0.042; Fig. 3a) and the short-term disease progression (HR = 1.989, 95%CI 1.209–3.271, *p* = 0.012; Fig. 3b) of the patients following first-line chemotherapy.

### 3′U-tRF^SerTGA^-fitted multivariate models improve patients’ risk-stratification and prognosis

**Fig. 4:**
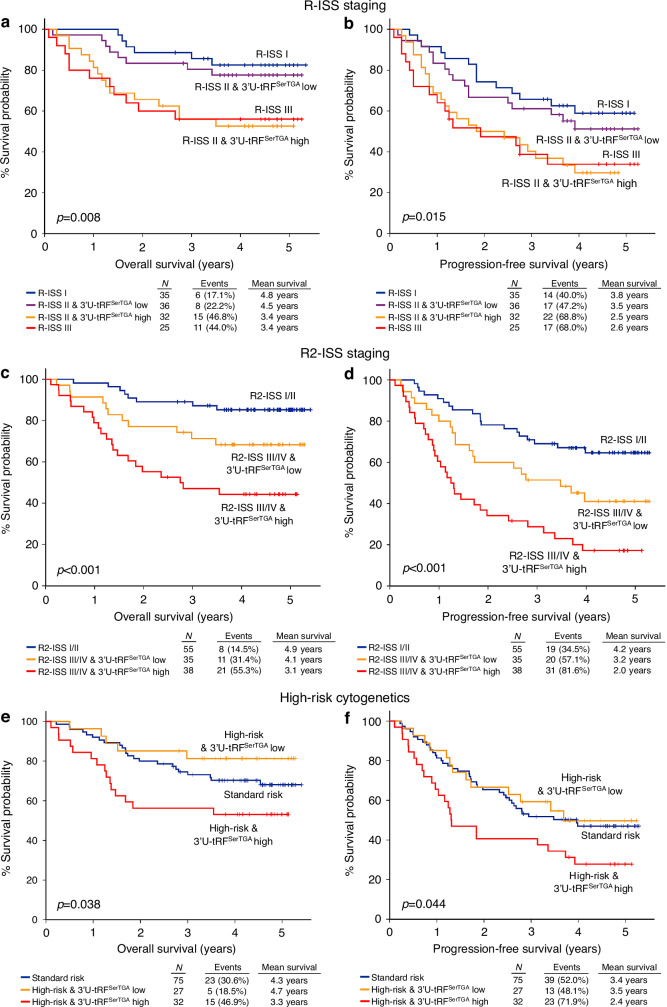
Evaluation of 3′U-tRF^SerTGA^ levels in CD138+ plasma cells improves risk-stratification and results to superior clinical benefit in MM prognosis. Kaplan–Meier survival curves for the OS (left) and PFS (right) of MM patients according to 3′U-tRF^SerTGA^ levels in CD138+ plasma cells in combination with R-ISS stage (**a**, **b**), R2-ISS stage (**c**, **d**) and high-risk cytogenetics (**e**, **f**). *p*-values calculated by log-rank test.

The independent prognostic value of 3′U-tRF^SerTGA^ in CD138+ plasma cells prompted us to assess the impact of 3′U-tRF^SerTGA^ on ameliorating the predictive value of the established and clinically used disease markers of R-ISS and R2-ISS staging, high-risk cytogenetics [t(4;14), t(14;16), del(17p13), gain/amp(1q21)] and response to first-line therapy. In this regard, the elevated 3′U-tRF^SerTGA^ levels at diagnosis were strongly associated with worse survival (*p* = 0.008; Fig. 4a) and short-term progression (*p* = 0.015; Fig. 4b) of R-ISS II patients, resembling the unfavourable prognosis of the R-ISS III group, while 3′U-tRF^SerTGA^-low R-ISS II patients presented similar outcome to R-ISS I group. Similarly, we have further classified R2-ISS III/IV based on 3′U-tRF^SerTGA^ levels, highlighting the worse prognosis of the R2-ISS III/IV patients with elevated levels of 3′U-tRF^SerTGA^ both for OS (*p* < 0.001; Fig. 4c) and PFS (*p* < 0.001; Fig. 4d).

**Fig. 5:**
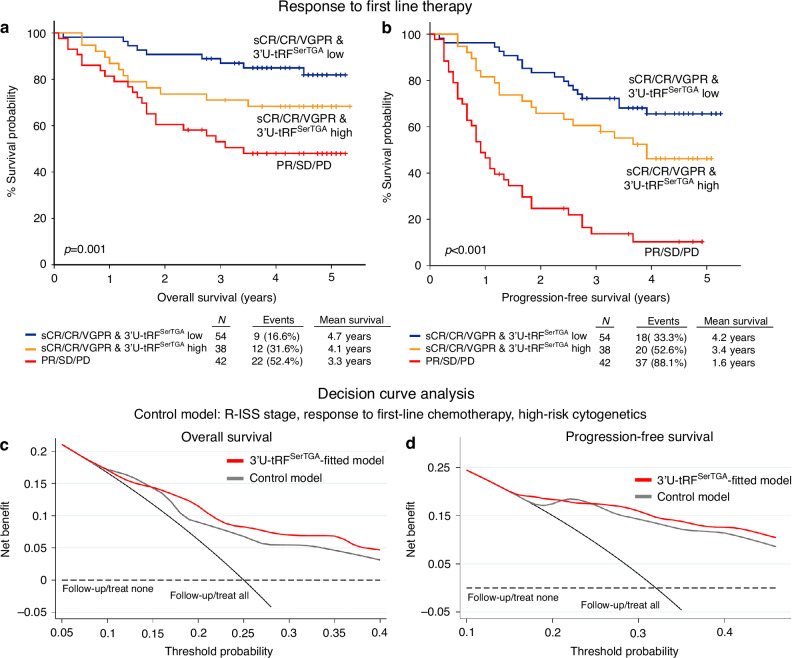
3′U-tRF^SerTGA^-fitted models enhance risk-stratification and provide superior clinical net benefit in MM prognostics. Kaplan–Meier survival curves for the OS (**a**) and PFS (**b**) of MM patients according to 3′U-tRF^SerTGA^ levels in CD138+ plasma cells in combination with response to first-line therapy. Decision curve analysis (DCA) curves of ‘3′U-tRF^SerTGA^-fitted’ and ‘control’ multivariate prognostic models for patients’ OS (**c**) and PFS (**d**). Net benefit is plotted against various ranges of threshold probabilities. sCR stringent complete response, CR complete response and VGPR very good partial response, PR partial response, SD stable disease, PD progressive disease.

Additionally, patients with high-risk cytogenetics and elevated 3′U-tRF^SerTGA^ levels at diagnosis displayed significantly shorter OS (*p* = 0.038; Fig. 4e) and PFS (*p* = 0.044; Fig. 4f) compared to ‘3′U-tRF^SerTGA^-low’ group which resemled the prognosis of standard-risk MM patients. Similarly, evaluation of 3′U-tRF^SerTGA^ levels with the response to first-line therapy ameliorated its predictive strength and resulted in a superior risk-stratification of MM outcome. In fact, patients with optimal responses (sCR, CR, VGPR) and elevated 3′U-tRF^SerTGA^ levels presented significantly worse OS (*p* = 0.001; Fig. 5a) and PFS (*p* < 0.001; Fig. 5b) compared to optimal responders within the 3′U-tRF^SerTGA^-low group. The abovementioned results are further maintained on the patients that have received bortezomib-lenalidomide-dexamethasone (74.3% of screening cohort; Supplementary Fig. 4). Ultimately, DCA curve analysis of 3′U-tRF^SerTGA^-fitted prediction models, incorporating 3′U-tRF^SerTGA^ levels with R-ISS, response to first-line therapy and high-risk cytogenetics, highlighted the improved clinical net benefit for patients’ OS (Fig. 5c) and PFS (Fig. 5d), compared to the control model of the clinical markers alone.

## Discussion

Although major clinical advancements have been made, MM remains incurable and most patients eventually develop drug resistance and experience disease relapse. Thus, deepening our understanding on disease molecular underpinnings and advancing new tools to improve patient prognosis and guide treatment decisions are of first clinical importance. Despite the fact that 3′U-tRFs have been recently oriented at RNA biology spotlight due to both their origin and deregulation in multiple cancer models [26, 27], research focusing on 3′U-tRFs and MM is still missing. Here, analysing small RNA-seq data, we have performed, for the first time, an integrative analysis of 3′U-tRFs in MM, highlighting 3′U-tRF^SerTGA^ clinical utility in ameliorating patients’ risk-stratification and prediction of treatment outcome.

Small RNA-seq data analysis demonstrated 3′U-tRF^SerTGA^ as the most abundant 3′U-tRF in malignant plasma cells and markedly elevated compared to normal plasma cells, while a first insight into 3′U-tRF^SerTGA^ mechanistic role unveiled the significant enrichment of target genes associated with localisation and regulation of gene signalling. Along the same lines, transfection of H929 and U266 with 3′U-tRF^SerTGA^ resulted in significantly increased cell growth rates, verifying its active implication in cell viability and growth of myeloma plasma cells and prompting us for a further thorough clinical evaluation on MM prognosis. Strikingly, the analysis of our screening cohort highlighted that elevated 3′U-tRF^SerTGA^ levels in CD138+ plasma cells at diagnosis are associated with significantly higher risk for short-term progression and poor survival of the patients following first-line chemotherapy, independently of the established disease markers and patients’ clinicopathological data, including R-ISS staging, high-risk cytogenetics, LDH and B2M levels and age. Additionally, 3′U-tRF^SerTGA^-fitted multivariate models significantly ameliorated risk-stratification and prognosis of the patients, resulting in advanced positive prediction of post-treatment outcome within the clinically heterogeneous group of R-ISS II patients. More precisely, R-ISS II patients with elevated 3′U-tRF^SerTGA^ levels were associated with short-term progression and worse survival, resembling the unfavourable prognosis of R-ISS III patients. Similarly, elevated 3′U-tRF^SerTGA^ translated to advanced positive prediction of poor treatment response of R2-ISS III/IV patients, high-risk cytogenetics and optimal responders to first-line chemotherapy groups.

Notably, our findings were in line with previous studies demonstrating the oncogenic function of 3′U-tRF^SerTGA^ in prostate cancer, where the upregulation of 3′U-tRF^SerTGA^ promoted the proliferation of prostate cancer cells, while its knockdown led to cell cycle arrest [28]. Similarly, Pekarsky et al. underlined 3′U-tRF^SerTGA^ as the most upregulated 3′U-tRF in aggressive chronic lymphocytic leukaemia (CLL) compared to indolent CLL [26], while Ballati et al. highlighted the upregulation of 3′U-tRF^SerTGA^ in carcinomas compared to adenomas, correlating 3′U-tRF^SerTGA^ with colon cells malignant transformation [27]. The elucidation of the mechanistic depth of 3′U-tRF^SerTGA^ in myeloma cells is intriguing, and surely our study paves the way and could stand as a scaffold for further future investigations. The absence of institutionally-independent validation cohort represents one of our study’s limitations, highlighting the need for future work to validate the prognostic significance and translational impact of 3′U-tRF^SerTGA^ in independent patient cohorts. Although neglected so far, recent evidence has increasingly highlighted tRFs as essential regulators of gene expression, playing roles as both oncogenes and tumour suppressors [29]. More precisely, 3′-tRF^ValCAC^ was documented to stimulate cell survival and migration in gastric cancer by inhibiting p53 signalling [30], while 5′h-tiRNA^ValCAC^ enhance pancreatic cancer cells’ metastasis via stimulating *c-MYC* transcription [31]. In a clinical perspective, increased levels of 5′-tRF^LysCTT^ and i-tRF^GlyGCC^ have been associated with adverse prognosis in bladder and ovarian cancer, respectively [32, 33], while in prostate cancer, 5′-tRF^LysCTT^/3′-tRF^PheGAA^ ratio was linked to poor survival outcome [34]. Regarding 3′U-tRFs, our group has demonstrated the ability of 3′U-tRF^ValCAC^ to enhance ovarian cancer cells’ growth and migration [35], while 3′U-tRF^SerGCT^ has been highlighted to stimulate proliferation in breast cancer [36]. Moreover, Veneziano et al. highlighted a potential protective role of specific 3′U-tRFs derived from tRNA^HisGTG^ in CLL [37], while 3′U-tRFs derived from tRNA^CysGCA^ suppressed glioma progression through direct VAV2 binding [38]. Intriguingly, tRFs implication in MM clinical management has been minimally reported, focusing mostly on tRFs derived from mature tRNAs [39].

Conclusively, our study aimed to evaluate 3′U-tRFs emergence and clinical impact in MM. Herein, we have highlighted the significant upregulation of 3′U-tRF^SerTGA^ in malignant compared to normal plasma cells, as well as the tumour-promoting functions of 3′U-tRF^SerTGA^ in MM cell growth. Additionally, we have unveiled the significantly higher risk for short-term progression and worse survival following first-line chemotherapy of the MM patients with elevated 3′U-tRF^SerTGA^ levels in CD138+ plasma cells at diagnosis, independently of clinicopathological data. Notably, 3′U-tRF^SerTGA^-fitted multivariate models strongly improved patients’ risk-stratification and prediction of treatment outcome compared to the routinely used clinically MM markers, serving personalised prognostics and precision medicine in MM.

## Supplementary information

Supplemental Figure 1

Supplemental Figure 2

Supplemental Figure 3

Supplemental Figure 4

Supplemental Table 1

## Data Availability

All the data are available from the corresponding authors on reasonable request.
