# Supplementary figures and images for: Delving into tRNA-derived small RNAs in multiple myeloma: elevated 3′U-tRFSerTGA leads to poor disease prognosis
Source: Br J Cancer. 2026 May 4;135(3):470–9. doi: 10.1038/s41416-026-03447-5 (PMC13373171; doi:10.1038/s41416-026-03447-5)

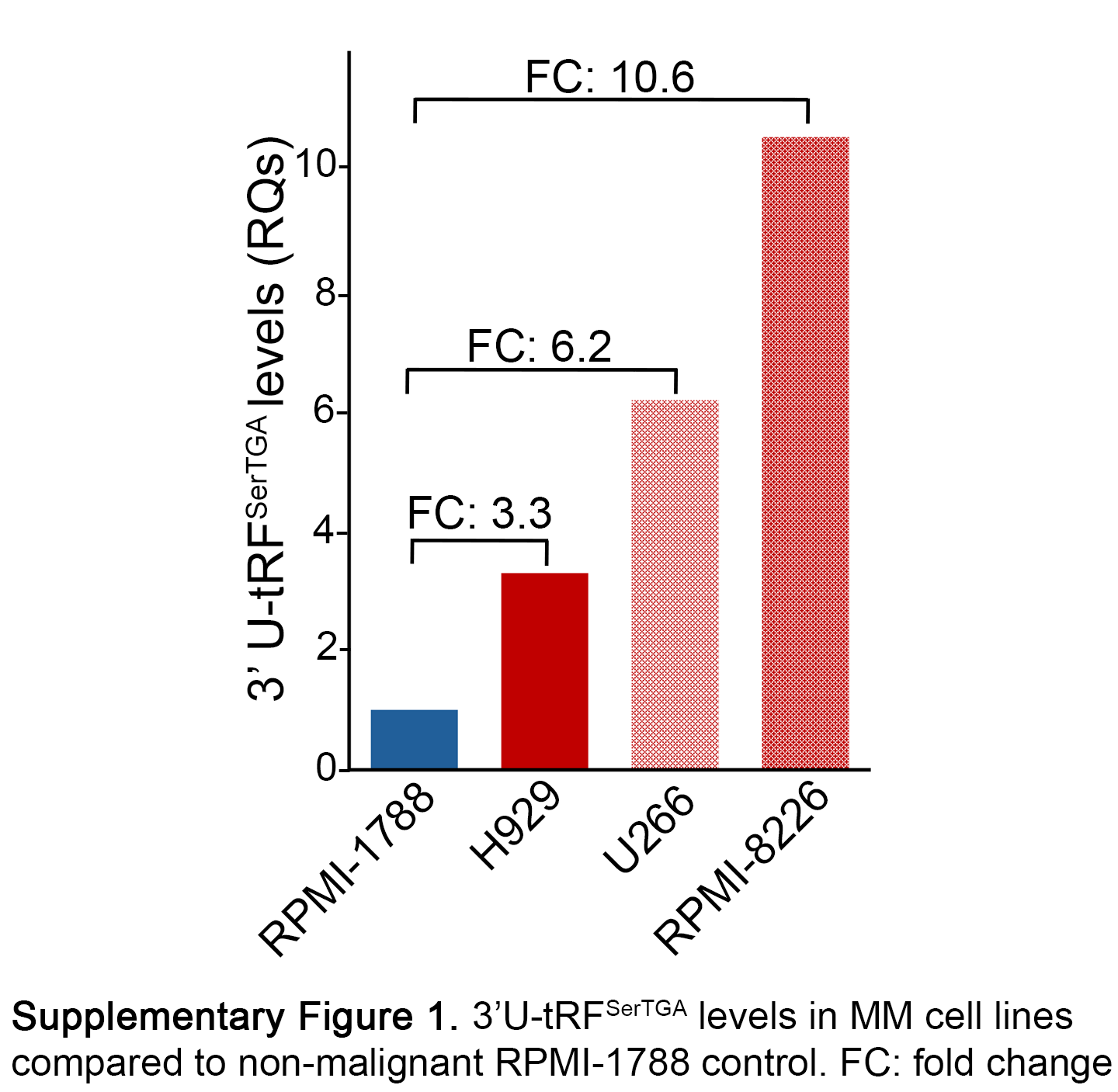

Supplement: Supplementary file 1 — Supplemental Figure 1 [file 41416_2026_3447_MOESM1_ESM.tif]

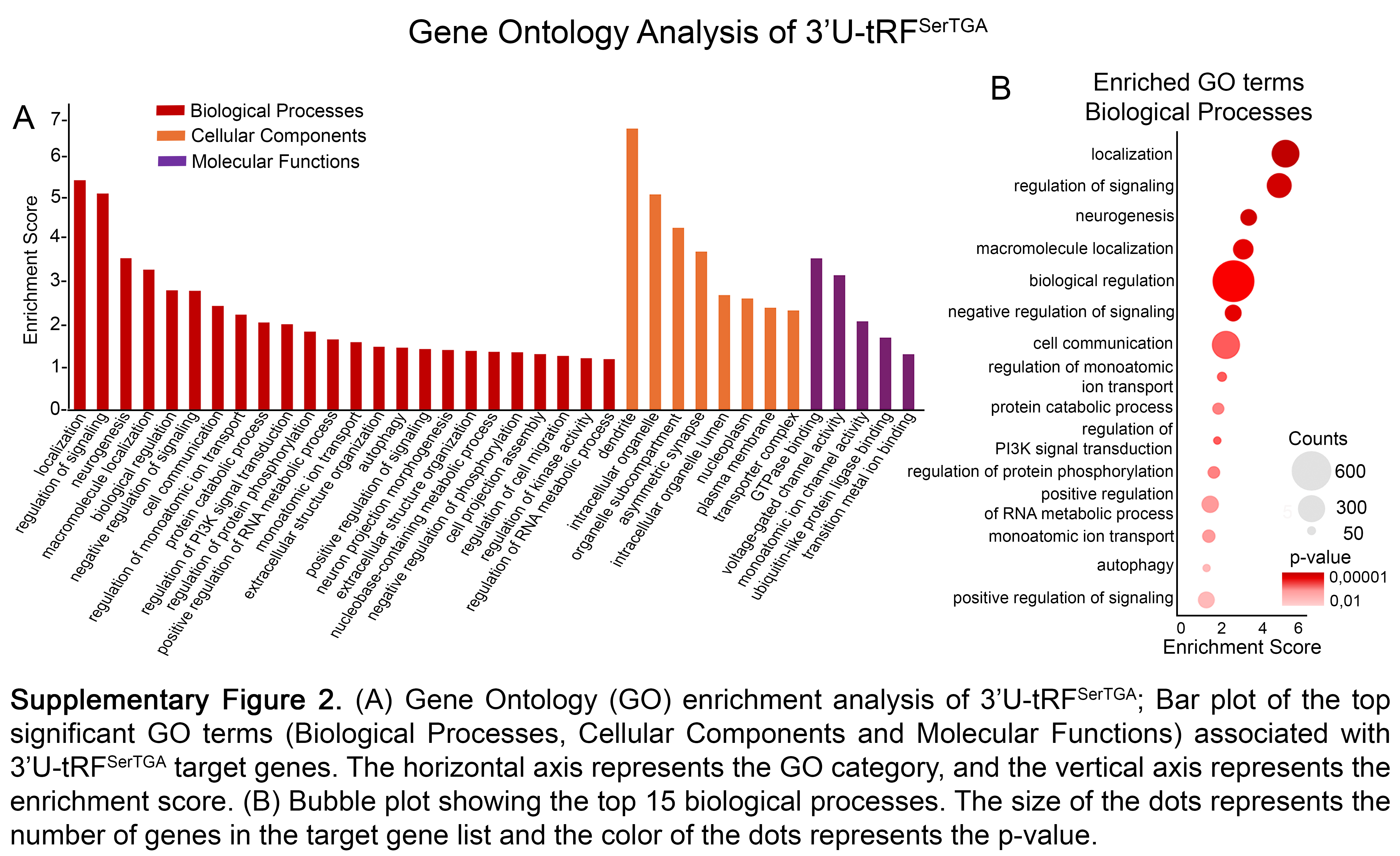

Supplement: Supplementary file 2 — Supplemental Figure 2 [file 41416_2026_3447_MOESM2_ESM.tif]

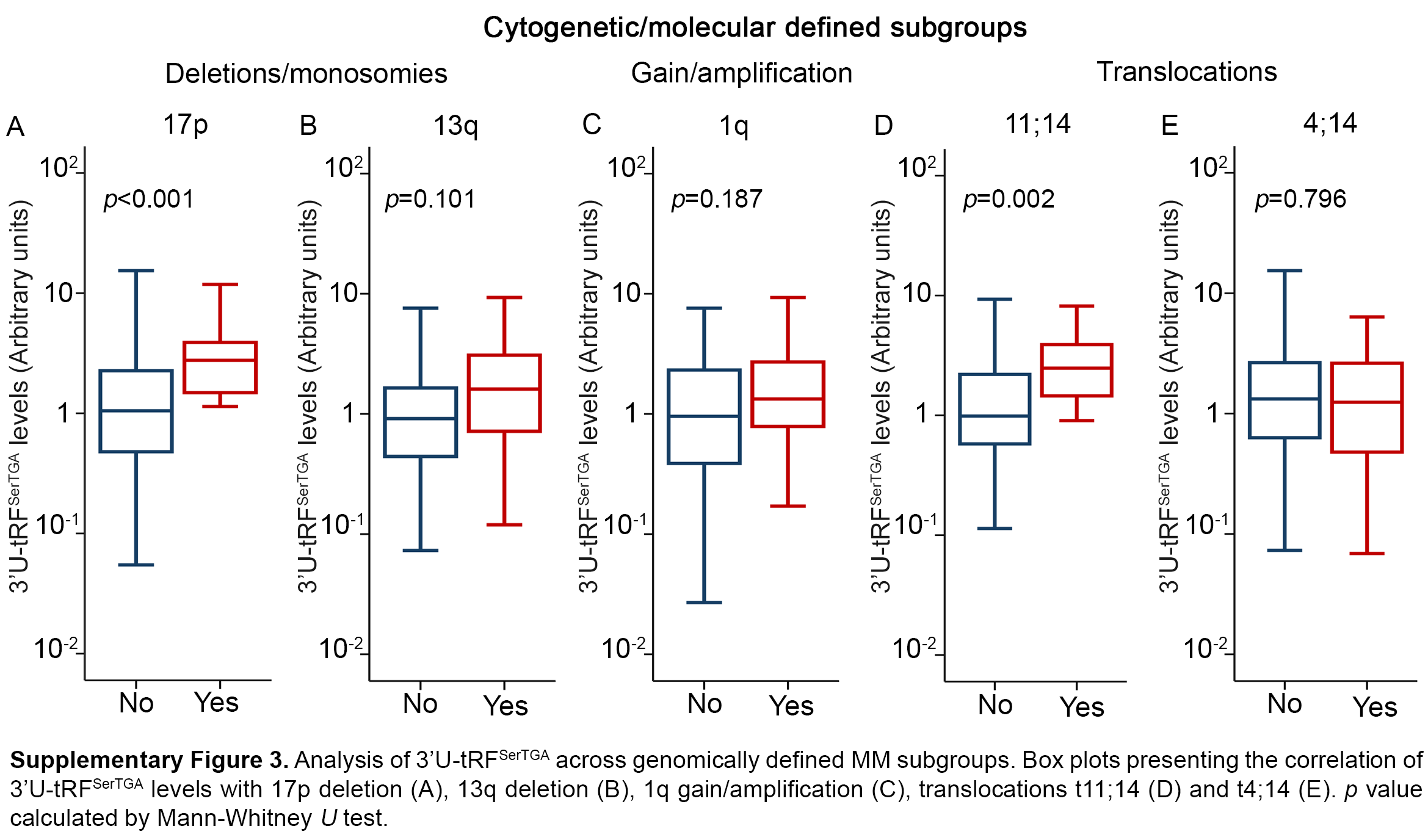

Supplement: Supplementary file 3 — Supplemental Figure 3 [file 41416_2026_3447_MOESM3_ESM.tif]

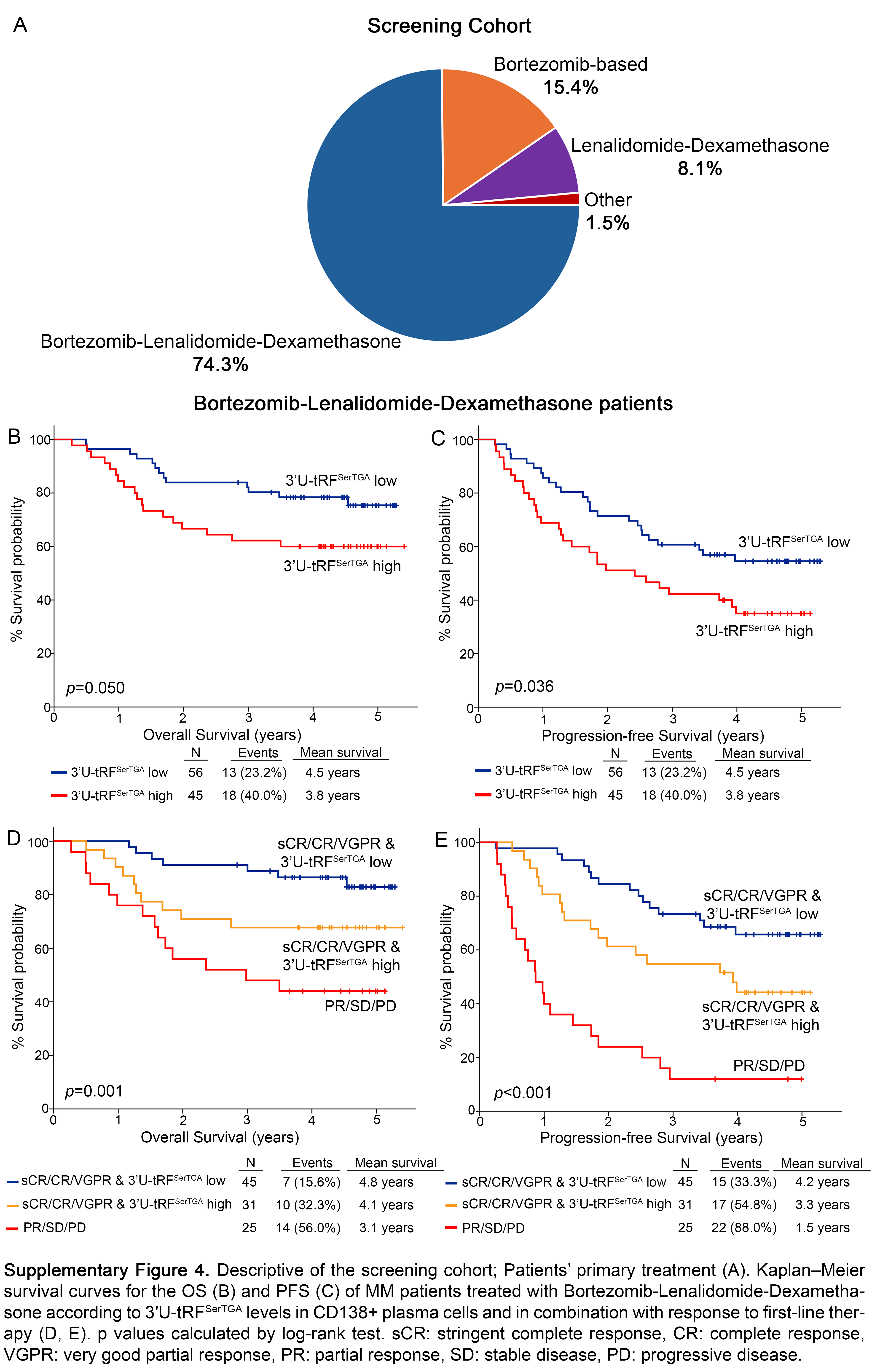

Supplement: Supplementary file 4 — Supplemental Figure 4 [file 41416_2026_3447_MOESM4_ESM.tif]
