# Supplementary material for: Delving into tRNA-derived small RNAs in multiple myeloma: elevated 3′U-tRFSerTGA leads to poor disease prognosis
Source: Br J Cancer. 2026 May 4;135(3):470–9. doi: 10.1038/s41416-026-03447-5 (PMC13373171; doi:10.1038/s41416-026-03447-5)
Supplement: Supplementary file 5 — Supplemental Table 1 [file 41416_2026_3447_MOESM5_ESM.docx]

**Suppl. Table 1.** Cox regression analysis for the prediction of MM patients’ risk for death (OS) and progression (PFS) based on CD138+ 3’U-tRF^SerTGA^ levels

|  | ***Univariate analysis*** | | | | | | | | | |
| --- | --- | --- | --- | --- | --- | --- | --- | --- | --- | --- |
|  | **Overall survival (OS)** | | | | | **Progression-free survival (PFS)** | | | | |
| **Covariant** | **HR^a^** | **95% CI^b^** | ***p*-value^c^** | **Bootstrap**  **BCa 95% CI^d^** | **Bootstrap**  ***p*-value^c^** | **HR^a^** | **95% CI^b^** | ***p*-value^c^** | **Bootstrap**  **BCa 95% CI^d^** | **Bootstrap**  ***p*-value^c^** |
| **CD138+ 3’U-tRF^SerTGA^**  Low expression  High expression | 1.00  1.972 | 1.081-3.599 | 0.027 | 1.070-3.962 | 0.026 | 1.00  1.703 | 1.087-2.669 | 0.020 | 1.095-2.723 | 0.019 |
| **R-ISS Stage**  R-ISS I / II  R-ISS III | 1.00  1.899 | 0.948-3.803 | 0.070 | 0.803-4.024 | 0.087 | 1.00  1.745 | 1.010-3.017 | 0.046 | 0.946-3.182 | 0.057 |
| **High-risk cytogenetics**  No  Yes | 1.00  1.211 | 0.665-2.206 | 0.531 | 0.635-2.221 | 0.514 | 1.00  1.348 | 0.856-2.121 | 0.197 | 0.838-2.132 | 0.203 |
| **LDH**  < 220 U/L  ≥ 220 U/L | 1.00  1.614 | 0.844-3.087 | 0.148 | 0.746-3.144 | 0.159 | 1.00  1.738 | 1.058-2.855 | 0.029 | 1.023-2.910 | 0.034 |
| **B2M**  < 5.5 mg/L  ≥ 5.5 mg/L | 1.00  2.491 | 1.363-4.551 | 0.003 | 1.332-4.868 | 0.002 | 1.00  2.185 | 1.387-3.444 | 0.001 | 1.334-3.482 | 0.001 |
| **Gender**  Female  Male | 1.00  1.178 | 0.645-2.148 | 0.594 | 0.639-2.142 | 0.577 | 1.00  0.818 | 0.521-1.283 | 0.381 | 0.527-1.337 | 0.379 |
| **Age** (continuous) | 1.054 | 1.025-1.084 | <0.001 | 1.029-1.087 | 0.001 | 1.031 | 1.009-1.052 | 0.005 | 1.007-1.054 | 0.007 |

|  | ***Multivariate analysis^e^*** | | | | | | | | | |
| --- | --- | --- | --- | --- | --- | --- | --- | --- | --- | --- |
|  | **Overall survival (OS)** | | | | | **Progression-free survival (PFS)** | | | | |
| **Covariant** | **HR^a^** | **95% CI^b^** | ***p*-value^c^** | **Bootstrap**  **BCa 95% CI^d^** | **Bootstrap**  ***p*-value^c^** | **HR^a^** | **95% CI^b^** | ***p*-value^c^** | **Bootstrap**  **BCa 95% CI^d^** | **Bootstrap**  ***p*-value^c^** |
| **CD138+ 3’U-tRF^SerTGA^**  Low expression  High expression | 1.00  1.923 | 0.997-3.710 | 0.051 | 1.025-4.189 | 0.042 | 1.00  1.989 | 1.209-3.271 | 0.007 | 1.186 -3.699 | 0.012 |
| **R-ISS Stage**  R-ISS I / II  R-ISS III | 1.00  0.602 | 0.219-1.654 | 0.325 | 0.152-3.012 | 0.453 | 1.00  0.638 | 0.283-1.437 | 0.278 | 0.233-1.802 | 0.354 |
| **High-risk cytogenetics**  No  Yes | 1.00  1.188 | 0.621-2.272 | 0.602 | 0.580-2.767 | 0.651 | 1.00  1.419 | 0.872-2.309 | 0.159 | 0.844-2.674 | 0.182 |
| **LDH**  ≤ 220 U/L  ≥ 220 U/L | 1.00  2.057 | 0.888-4.762 | 0.092 | 0.586-6.952 | 0.178 | 1.00  2.089 | 1.113-3.923 | 0.022 | 0.970-4.585 | 0.041 |
| **B2M**  ≤ 5.5 mg/L  ≥ 5.5 mg/L | 1.00  2.431 | 1.097-5.386 | 0.029 | 0.970- 6.681 | 0.038 | 1.00  2.393 | 1.280-4.471 | 0.006 | 1.158-5.178 | 0.011 |
| **Gender**  Female  Male | 1.00  1.130 | 0.595-2.144 | 0.709 | 0.528-2.634 | 0.747 | 1.00  0.803 | 0.498-1.294 | 0.803 | 0.488-1.402 | 0.398 |
| **Age** (Continuous) | 1.045 | 1.013-1.079 | 0.006 | 1.013-1.101 | 0.016 | 1.022 | 0.998-1.047 | 0.070 | 0.996-1.054 | 0.105 |

a: Hazard Ratio, b: 95% confidence interval of the estimated HR, c: Bootstrap *p*-value is based on 1000 bootstrap samples d: Bootstrap bias-corrected and accelerated 95% CI of the estimated HR based on 1000 bootstrap samples, e: Multivariate analysis adjusted for CD138+ 3’U-tRF^SerTGA^ levels, R-ISS, high-risk cytogenetics, B2M / LDH, gender and age.
